# Supplementary figures and images for: Size Shapes the Active Microbiome of the Methanogenic Granules, Corroborating a Biofilm Life Cycle
Source: mSystems. 2020 Sep 29;5(5):e00323-20. doi: 10.1128/mSystems.00323-20 (PMC7527134; doi:10.1128/mSystems.00323-20)

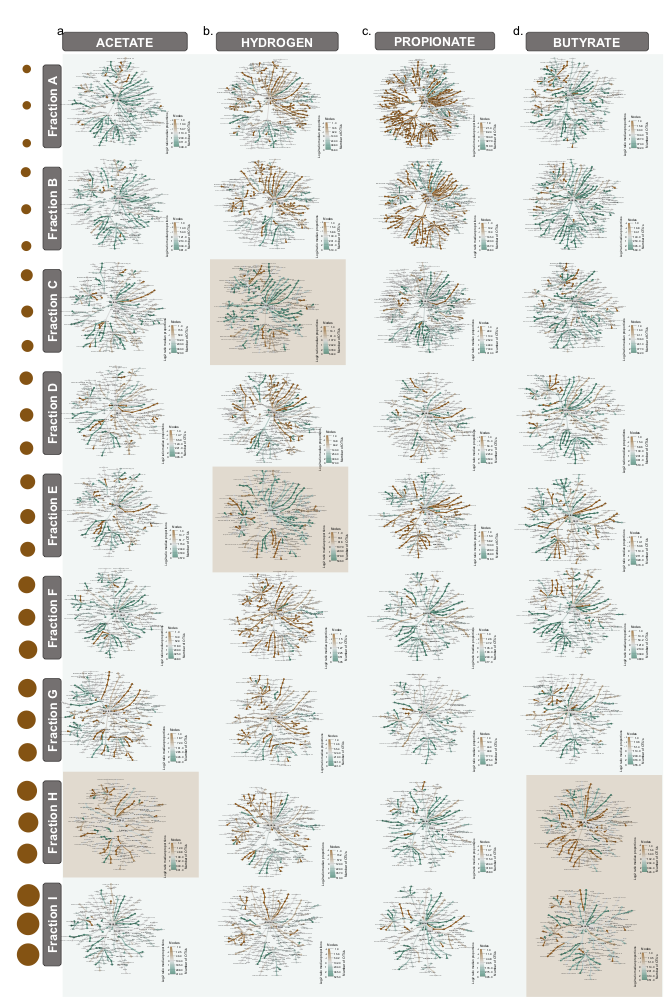

Supplement: FIG S1 [file mSystems.00323-20-sf001.tif]

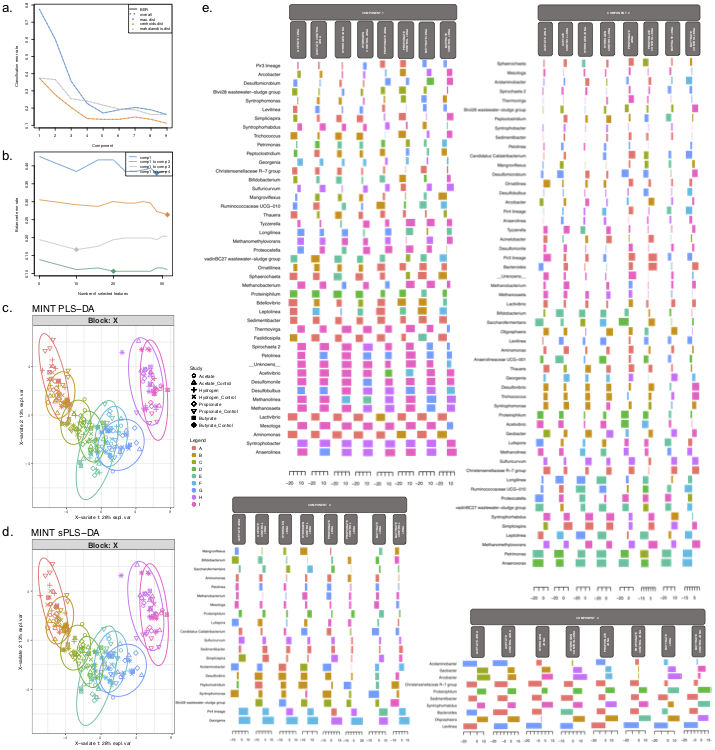

Supplement: FIG S2 [file mSystems.00323-20-sf002.tif]

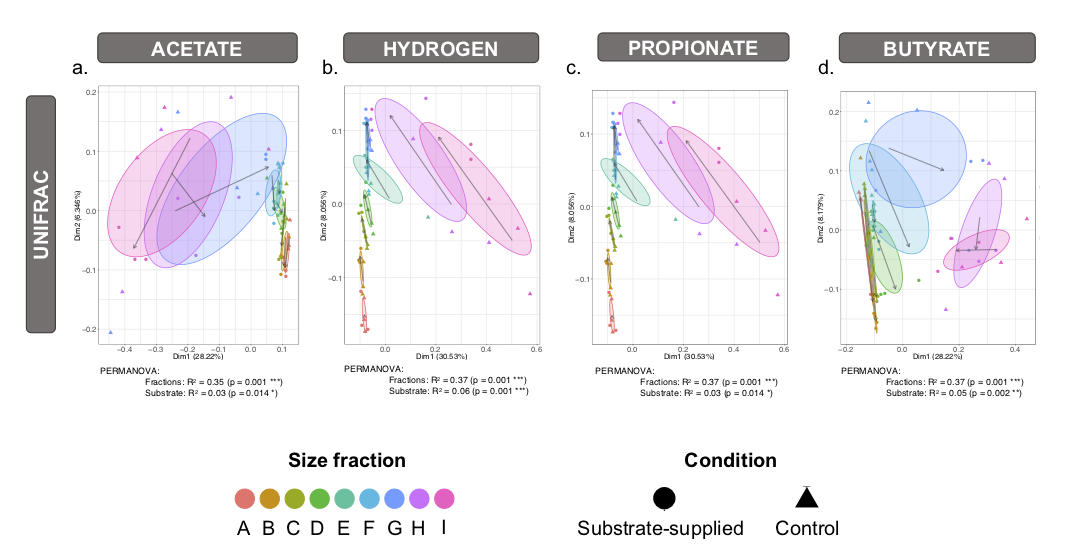

Supplement: FIG S3 [file mSystems.00323-20-sf003.tif]
